# Effectiveness and safety of auricular therapy for post-stroke depression: A protocol for systematic review and meta-analysis

**DOI:** 10.1097/MD.0000000000028505

**Published:** 2022-01-14

**Authors:** Mingzhi Tang, Sisi Feng, Yihao Zhou, Wenjing Zhang, Yu Wang, Dan Feng, Yong Qin, Yang Chen, Yanan Hu, Haijing Liu

**Affiliations:** Yunnan University of Traditional Chinese Medicine, Kunming, China.

**Keywords:** auricular therapy, meta-analysis, post-stroke depression, protocol, systematic review

## Abstract

**Background:**

Post-stroke depression is a common and serious complication after stroke. Its main clinical manifestations are depression or instability, loss of interest, loss of appetite, sleep disorders, pessimism, and unworthiness, and even suicidal tendencies. Auricular therapy (AT), as part of traditional Chinese acupuncture, has achieved good results in the treatment of depression, but different clinical studies have shown mixed results. Therefore, the aim of this systematic review is to assess the effectiveness and safety of AT for post-stroke depression.

**Methods:**

Two reviewers will electronically search the following databases: the Cochrane Central Register of Controlled Trials; Medline (via PubMed); Excerpt Medica Database; China National Knowledge Infrastructure; Chinese Biomedical Literature Database; Chinese Scientific Journal Database; and Wan–Fang Database from the inception to January 1, 2022. Study selection, data extraction, and assessment of study quality will be performed independently by 2 reviewers. If it is appropriate for a meta-analysis, Review Manager Version 5.3 statistical software will be used; otherwise, a descriptive analysis will be conducted. Data will be synthesized by either the fixed-effects or random-effects model according to a heterogeneity test. The results will be presented as risk ratio with 95% confidence intervals for dichotomous data and weight mean difference or standard mean difference 95% confidence intervals for continuous data.

**Result:**

This study will provide a comprehensive review of the available evidence for the treatment of AT with post-stroke depression.

**Conclusions:**

The conclusions of our study will provide an evidence to judge whether AT is an effective and safe intervention for patients with post-stroke depression.

**Trial registration number:**

PROSPERO CRD42021289870.

## 1 Introduction

### 1.1 Description of the condition

Stroke is the leading cause of death, disability, and shortened life span worldwide, and its incidence and prevalence increase with age. Post-stroke depression (PSD) is the most common psychiatric disorder after stroke, negatively affecting functional outcome, recovery response, and quality of life in survivors, with approximately one third affected by PSD.^[1]^ The fatality rate of PSD was 1.28 to 1.75 times higher than that of stroke alone.^[2]^ Its main clinical manifestations are depression or instability, loss of interest, loss of appetite, sleep disorders, pessimism, and unworthiness, and even suicidal tendencies. Recently, a thorough meta-analysis of 108 studies on mood disorders observed 147 cases from 2 days to 7 years post-stroke and demonstrated a 33.5% prevalence of any depressive disorder. Major Depression accounted for 17.7%, Minor Depression for 13.2%, anxiety disorder for 9.8%, adjustment disorder for 6.9% and dysthymia for 3.1%.^[3]^

The pathophysiology of PSD is multifactorial and likely involves decreased levels of monoamines, abnormal neurotrophic response, increased inflammation with dysregulation of hypothalamic-pituitary-adrenal axis, and glutamate-mediated excitotoxicity.^[4]^ Depression before stroke, physical disability, cognitive impairment, and stroke severity are the most consistent predictors of PSD.^[5]^

### 1.2 Description of the intervention

As an important part of traditional Chinese acupuncture, auricular therapy (AT) is a method for diagnosing and treating physical and psychosomatic dysfunctions by stimulating a specific point in the ear,^[6]^ including acupuncture, electroacupuncture, acupressure, drug injection, electric pulse stimulation, moxibustion, auricle bloodletting, etc. AT has been employed for approximately 2500 years, for which the oldest record is *Huang Di Nei Jing—the Yellow Emperor's Classic of Internal Medicine*, written in Chinese, and a report by Hippocrates is the oldest Western record.^[7]^ In 1990, the World Health Organization recognized auricular acupuncture as a microacupuncture system that can have a positive impact on regulating whole-body function.^[8]^ In recent decades, on the basis of inheriting the traditional auricular diagnosis methods and absorbing the relevant theories and principles of modern medicine, the AT has been refined and perfected, gradually forming the current auricular diagnosis and treatment system, which has been applied in clinical diagnosis and treatment and experiments.

### 1.3 How the intervention might work

Traditional medicine believes that the ear and the meridians, viscera have a close connection, the human ear acupuncture points corresponding to the body of multiple important organs, when the human body some viscera dysfunction, the corresponding parts of the ear will produce color changes, blood vessel changes, and other changes, but also in the formation of pain points in the relevant ear. Therefore, various related diseases can be treated by stimulating specific acupuncture points on the ear that represent specific parts or organs of the body. In the auricular system of modern medicine, the ear is innervated by cranial and spinal nerves, which are separated into motor and sensory areas. Nogier designed a map of inverted fetuses based on the distribution of auricular reflex points and he believed that the relationship between AT and the areas of the body is due to the vagus nerveautonomic nervous system.^[9]^ Some studies have shown that AT has achieved good efficacy in the treatment of depression, and the mechanisms may including directly and indirectly modulating the activity and connectivity of key brain regions involved in depression and mood regulation; inhibiting neuro-inflammatory sensitization; modulating hippocampal neurogenesis; regulating the microbiome brain gut axis; and suppressing the vagal nerve inflammatory responses.^[10,11]^

### 1.4 Why it is important to do this review

In the treatment of PSD, antidepressants such as citalopram, escitalopram, nortriptyline, milnacipran, mirtazapine, piracetam, and fluoxetine are most commonly used.^[12]^ However, adverse effects of antidepressants were evident, including blurred vision, urinary retention, low blood pressure, sexual dysfunction, tremors, and severe insomnia.^[13]^ And 1 study showed that up to 60% of patients do not respond adequately to antidepressant treatment.^[14]^ Therefore, it is necessary to explore effective and safe methods for the treatment of PSD. AT, as part of traditional Chinese acupuncture, has achieved good results in the treatment of depression, but different clinical studies have shown mixed results. Through review, there have been no systematic reviews that have been designed specifically to investigate the efficacy of AT for PSD. Therefore, we hope to conduct a systematic evaluation of the published literature, with the aim of exploring the Effectiveness and safety of AT in the treatment of PSD, and to draw more reliable conclusions through meta-analysis to provide concluding evidence for clinical practice.

## 2 Methods and analysis

### 2.1 Study registration

This study protocol has been registered in the international prospective register of systematic reviews (PROSPERO) with a trial registration number CRD42021289870, and will adhere to the guidelines of the Preferred Reporting Items for Systematic Reviews and Meta-Analysis Protocols statement (PRISMA-P).^[15]^

### 2.2 Ethics and communication

This type of study is systematic reviews, and the entire study process does not involve the privacy information of individual patients, therefore does not require ethical approval.

### 2.3 Inclusion and exclusion criteria

#### 2.3.1 Types of studies

All randomized controlled trials will be included, without restrictions on publication status, but the language will be limited to English and Chinese. Other type studies including reviews, animal experiments, theory discussion, case reports, conference articles, letters to editor and non-randomized controlled trials study will be excluded.

#### 2.3.2 Types of participants

This study will include participants with PSD, regardless of gender, age, occupation, education, and severity, etc. Patients with primary depression will be excluded.

#### 2.3.3 Types of interventions

Research using AT or AT combined with other therapies, without limiting of acupoints and courses of treatment.

#### 2.3.4 Types of comparisons

The control group's treatment is not limited, including no treatment, placebo, or any control considered for comparison in a single systematic review.

#### 2.3.5 Types of outcome measures

##### 2.3.5.1 Primary outcome

Hamilton Depression Rating Scale.

##### 2.3.5.2 Secondary outcomes

Self-rating Depression Scale, total effective rate, cure rate, and adverse reactions.

### 2.4 Data sources and search strategies

The following databases from the inception to January 1, 2022 will be searched by 2 independent reviewers, without restriction to publication status: the Cochrane Central Register of Controlled Trials; Medline (via PubMed); Excerpt Medica Database; China National Knowledge Infrastructure); Chinese Biomedical Literature Database; Chinese Scientific Journal Database; and Wan–Fang Database. A search strategy for Medline (via PubMed) database, which is established according to the Cochrane handbook guidelines, is shown in Table 1. Similar search strategies will be applied for the other databases. Before this review completed, the 2 reviewers will conduct the searching once again to ensure the latest studies could be included.

**Table 1:** The search strategy for PubMed.

Order	Strategy
#1	Search: “Stroke”[Mesh]
#2	Search: “Strokes”[Title/Abstract] OR “Cerebrovascular Accident”[Title/Abstract] OR “Brain Vascular Accident”[Title/Abstract] OR “Cerebrovascular Stroke”[Title/Abstract] OR “Apoplexy”[Title/Abstract] OR “Cerebral Stroke”[Title/Abstract]
#3	#1 OR #2
#4	Search: “Depression”[Mesh]
#5	Search: “Depressions”[Title/Abstract] OR “Depressive Symptom”[Title/Abstract] OR “Emotional Depression”[Title/Abstract] OR “Post-stroke Depression”[Title/Abstract] OR “PSD“[Title/Abstract]
#6	Search: “Depressive Disorder”[Mesh]
#7	Search: “Depressive Disorders”[Title/Abstract] OR “Neurosis, Depressive”[Title/Abstract] OR “Depression, Endogenous”[Title/Abstract] OR “Depressive Syndrome”[Title/Abstract] OR “Neurotic Depression”[Title/Abstract] OR “Melancholia”[Title/Abstract] OR “Unipolar Depression”[Title/Abstract]
#8	#4 OR #5 OR #6 OR #7
#9	Search: “auricular”[Title/Abstract] OR “auricular therapy”[Title/Abstract] OR “auricular acupuncture”[Title/Abstract] OR “auricular acupoints”[Title/Abstract] OR “auricular point sticking”[Title/Abstract] OR “electric pulse stimulation”[Title/Abstract] OR “auricular massage”[Title/Abstract] OR “auricular bleed”[Title/Abstract]
#10	Search: “randomized controlled trial”[Publication Type] OR “RCT randomized controlled”[Publication Type] OR “random allocation”[Title/Abstract] OR “allocation, random”[Title/Abstract] OR “randomized, controlled”[Title/Abstract] OR “clinical trial”[Title/Abstract]
#11	Search: “humans”[MeSH Terms] NOT “animals”[MeSH Terms]
#12	#10 AND #11
#13	#3 AND #8 AND #9 AND #12

### 2.5 Selection of studies

**Figure 1:**
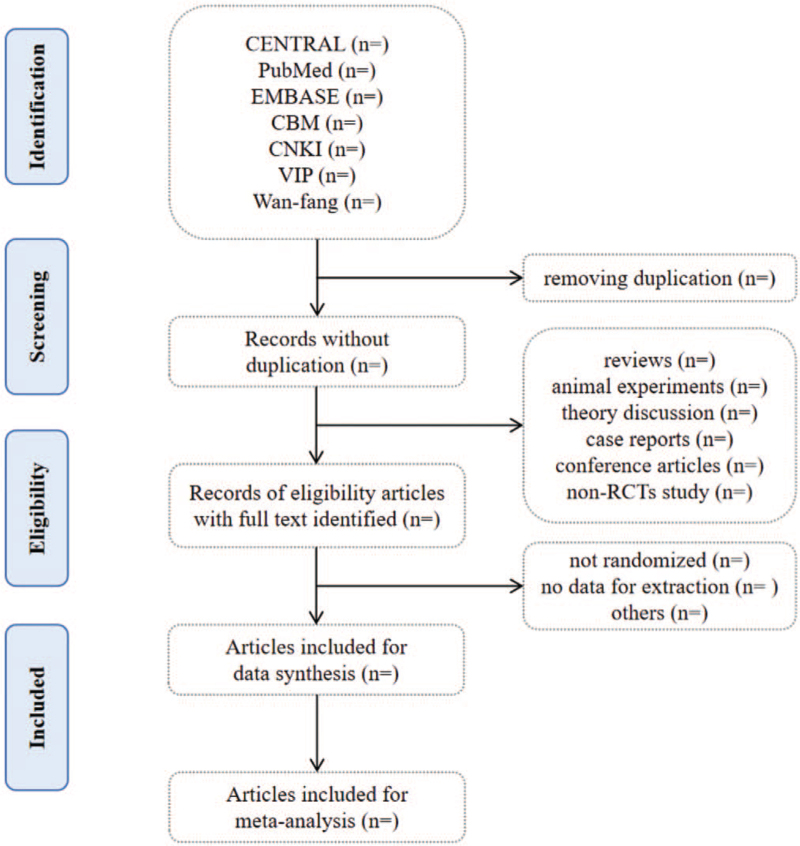
Flowchart of literature selection.

We plan to conduct this systematic review between January 1, 2022 and December 30, 2022. All reviewers have undergone a training to ensure a basic understanding of the background and purpose of the review. After electronic searching, the records will be uploaded to a database set up by EndNote software (V.X7). Records selected from other sources will also be moved to the same database. Two reviewers (MZT and SSF) will independently screen the titles, abstracts, and keywords of all retrieved studies and decide which trials meet the inclusion criteria. We will obtain the full text of all possibly relevant studies for further assessment. Excluded studies will be recorded with explanations. Any disagreements will be resolved by discussion between the 2 reviewers (MZT and SSF) and the third author (YHZ) for arbitration when necessary. We will contact reviewers of trials for clarification when necessary. The study flow diagram is shown in Figure 1.

### 2.6 Data extraction and management

A unified data extraction form will be designed by all of the reviewers and 2 reviewers (MZT and SSF) will then independently extract data in the following domains: general information, participants, methods, interventions, outcomes, and other information. Any disagreement will be discussed between the 2 reviewers, and further disagreements will be arbitrated by the third author (YHZ).

### 2.7 Dealing with missing data

We will attempt to contact the first or corresponding author included in the study by telephone or email to obtain missing or insufficient trial data. If missing data are unavailable, we will make an assumption using the terms “missing at random” and “not missing at random” to represent different scenarios, which is recommended in the Cochrane Handbook.^[16]^ For the data “missing at random”, only the available data will be analyzed. For the data “not missing at random”, we will displace the missing data with replacement values and a sensitivity analysis will be used to determine whether the results are inconsistent.

### 2.8 Assessment of risk of bias in included studies

The risk of bias will be assessed by 2 reviewers (WJZ and YW) with the Cochrane Collaborations tool for risk of bias assessment. The risk of bias in included studies will be evaluated according to the following aspects: sequence generation, allocation sequence concealment, blinding of participants and personnel and outcome assessors, incomplete outcome data, selective outcome reporting, and other sources of bias. The assessments will be classified into 3 levels: low risk, high risk, and unclear risk.

### 2.9 Data analysis and synthesis

We will use Review Manager Version 5.3 statistical software for data analysis and quantitative data synthesis. For continuous data, we will use standard mean difference to measure the treatment effect with 95% confidence intervals. For dichotomous data, a risk ratio with 95% confidence intervals for analysis will be adopted. On the basis of the data analysis, random effect or fixed effect models will be employed according to the heterogeneity given by *I*^2^ statistic value. To be concrete, a fixed effect model will be adopted if the heterogeneity is indicated as high (*I*^2^ < 50%); otherwise, a random effect model will be applied on the contrary. Additionally, if heterogeneity is considered significant, the subgroup or sensitivity analysis will be performed to distinguish the source of it. When it comes to the situation that the data are insufficient for quantitative analysis, we will only perform a descriptive analysis.

### 2.10 Subgroup analysis

If the result shows high heterogeneity, we will perform subgroup analysis based on the type of AT intervention, severity of depression, treatment duration, and other relevant parameters to explore potential sources of heterogeneity.

### 2.11 Sensitivity analysis

We will perform sensitivity analysis by reevaluating methodological quality, study types, sample size, missing data or other possible factors to validate the robustness of the primary results. If the difference is significant, we will explain carefully.

### 2.12 Assessment of reporting biases

If more than 10 studies are included in the meta-analysis, we will use funnel plot and Egger regression test to evaluate reporting bias.

### 2.13 Grading the quality of evidence

The Grading of Recommendations Assessment, Development, and Evaluation working group methodology will be applied for the quality of evidence for all outcomes.^[17]^ Six domains will be assessed, containing risk of bias, consistency, directness, precision, publication bias, and additional points. The assessments will be categorized into 4 levels: high, moderate, low, or very low.

## 3 Discussion

PSD is a series of emotional disorders syndrome after stroke, with a high incidence and an increasing trend year by year. It is one of the common and treatable complications after stroke. Antidepressants are the most commonly used treatment, but clinical studies showed obvious side effects and some patients do not respond adequately to them. Acupuncture, as an important part of traditional Chinese medicine, is one of the most popular non-drug therapies in the West and has been increasingly used in the clinical treatment of PSD. A systematic review and meta-analysis suggested that acupuncture is an effective and safe treatment for PSD.^[18]^ AT is an important part of traditional Chinese acupuncture and moxibustion, but there is a large difference between AT and acupuncture, which requires additional evaluation. Our study will generate evidence for AT in the treatment of PSD and help to reduce the uncertainty about the effectiveness of PSD management. The results will encourage further suggestions for AT clinical practice or guideline, which will draw wide attention.

## Author contributions

**Conceptualization:** Mingzhi Tang, Haijing Liu.

**Data curation:** Mingzhi Tang, Sisi Feng, Yihao Zhou, Wenjing Zhang, Yu Wang, Dan Feng.

**Formal analysis:** Mingzhi Tang, Yihao Zhou.

**Investigation:** Haijing Liu, Sisi Feng, Wenjing Zhang, Yong Qin.

**Methodology:** Mingzhi Tang, Sisi Feng, Yihao Zhou.

**Software:** Mingzhi Tang, Sisi Feng.

**Supervision:** Haijing Liu, Yang Chen, Yanan Hu.

**Writing – original draft:** Mingzhi Tang, Yihao Zhou.

**Writing – review & editing:** Haijing Liu.
